# Onset of Motor Complications and Medication Dose in Newly Diagnosed and Treated Parkinson’s Disease

**DOI:** 10.1155/padi/8827381

**Published:** 2026-07-31

**Authors:** Yasushi Osaki, Yukari Morita, Sho Ohtsuru, Tomohiro Shogase, Daiji Yoshimoto, Tatsuya Ikeda, Sayomi Kabeya, Yu Hashimoto, Takuya Matsushita

**Affiliations:** ^1^ Department of Neurology, Kochi Medical School Hospital, Kohasu Oko-cho, Nankoku-shi 785-8505, Kochi, Japan

**Keywords:** dyskinesias, levodopa, Parkinson’s disease

## Abstract

**Background:**

Parkinson’s disease (PD) patients under medical treatment experience motor complications (MCs), namely, ON/OFF fluctuations (ON/OFF) and dyskinesias.

**Methods:**

We assessed 120 newly diagnosed PD patients after the initiation of medical treatment who were followed up for at least 24 months. We reviewed the latency of ON/OFF or dyskinesias by months and calculated the levodopa dose (LD), levodopa dose divided by daily intake (LDdiv), and levodopa equivalent dose (LED). We estimated cumulative incidence and median latency by Kaplan–Meier survival analysis. We classified patients with MCs into four groups and patients with ON/OFF or dyskinesias into three groups, according to latency.

**Results:**

Seventy‐six patients experienced MCs with a median latency of 28 months. Fifty‐seven patients experienced ON/OFF and 19 experienced dyskinesias with a median latency of 28 and 50 months, respectively. The medication dose at which each patient experienced ON/OFF or dyskinesias varied widely. The median LD, LDdiv, and LED was 300 (237.5–350), 100 (79–111), and 300 (250–450) mg at the onset of MCs; 300 (200–300), 100 (83–125), and 300 (249–400) mg at the onset of ON/OFF; and 400 (375–500), 125 (105–133), and 600 (466–722.75) mg at the onset of dyskinesias, respectively. Consistently, the shortest latency group had the lowest medication dose, while the longest latency group had the highest. Five patients experienced dyskinesias before ON/OFF, with similar medication doses at the onset of ON/OFF and dyskinesias. Some patients experienced ON/OFF but have not yet developed dyskinesias under a comparable medication dose as patients who experienced dyskinesias; the latter had a significantly younger age at onset.

**Conclusions:**

The pathophysiological backgrounds and changes causing ON/OFF or dyskinesias are not the same for all patients. The presence of ON/OFF and higher medication doses does not always cause dyskinesias. A younger age at onset may be associated with the occurrence of dyskinesias.

## 1. Introduction

Patients with Parkinson’s disease (PD) suffer progressive disability from cardinal motor symptoms, motor complications (MCs), poorly levodopa‐responsive motor symptoms, and nonmotor symptoms [1, 2]. In our previous study, we observed the occurrence of MCs and gait problems and discussed that the latter was partly due to age‐related changes [3]. Further, dopaminergic medication may impair cognitive function, and this may lead to gait problems [3]. Apart from gait problems, even if physicians prescribe and titrate medications gradually to avoid MCs, they develop in most patients, namely ON/OFF fluctuations or dyskinesias, during the disease course.

Levodopa‐induced dyskinesias in PD include peak‐dose and diphasic dyskinesias. Regarding peak‐dose dyskinesias, the pathogenic mechanisms at the striatal level include the pulsatile stimulation of dopamine receptors, excessive presynaptic swing of dopamine leading to increased receptor occupancy, dissociation between low intrastriatal dopamine and high plasma and extracellular L‐dopa, downstream changes in the postsynaptic compartment, and abnormalities in nondopaminergic neurotransmitters. These events cause changes in the firing patterns and oscillatory activity between the basal ganglia and motor cortex, leading to the excessive disinhibition of thalamocortical neurons and overactivation of the motor cortex [4]. Conversely, diphasic dyskinesias are low‐dose phenomena that typically appear at the onset and end of levodopa antiparkinsonian action. Typical “off”‐state beta activity disappears with the onset of diphasic dyskinesias, whereas gamma activity is absent or minimal until their end. Theta activity during diphasic dyskinesias is similar to that observed during peak‐dose dyskinesias [5].

The prevalence of MCs and the medication dose at their onset have been reported [6–10], including clinical studies in which the patients were randomized and allocated medications [11, 12]. However, there are a few reports from a nonrandomized setting in which physicians prescribe and titrate medications from the introduction of treatment [13]. Here, we observed the onset of MCs and calculated the medication dose at onset in patients we diagnosed as de novo PD and followed up for at least 24 months.

## 2. Materials and Methods

### 2.1. Patient Population

We included 120 patients (60 men and 60 women) who were diagnosed with PD and started to receive treatment between May 2014 and November 2024 and were followed up regularly for at least 24 months. A flow diagram of the study is shown in Supporting Figure 1. For patients with MCs, we defined the endpoint as the time of the onset of either ON/OFF fluctuations or dyskinesias. For patients without MCs, we defined the endpoint as November 30, 2024. We diagnosed PD using the Movement Disorders Society Clinical Diagnostic Criteria for PD [14]. We excluded patients if they presented with significant memory impairment (Mini‐Mental State Examination score < 24) or dementia with Lewy bodies [15]. The median age at onset was 72 (interquartile range: 64–77) years.

This retrospective study was approved by the Ethics Committee of the Faculty of Medicine, Kochi University (approval no. 2023‐52). Informed consent was waived as the study involved only the retrieval of descriptions and data from medical records.

### 2.2. Clinical Assessments

We reviewed the occurrence of clinical symptoms, ON/OFF fluctuations, and dyskinesias in each patient at each visit and calculated the latencies of each symptom by month. We considered that patients had ON/OFF fluctuations or dyskinesias if the Movement Disorder Society‐sponsored revision of the Unified PD Rating Scale Part IV 4.3 or 4.1 score was ≥ 1, respectively [16]. PAR granted permission for the use of the Mini‐Mental State Examination scale in this study.

### 2.3. Medication Dose

We reviewed the medication dose of each patient. The prescribed drugs included levodopa, entacapone, opicapone, selegiline (oral), rasagiline, pramipexole (immediate‐release), pramipexole (extended‐release), ropinirole, ropinirole patch, rotigotine patch, amantadine hydrochloride (immediate‐release), zonisamide, trihexyphenidyl, and istradefylline. To calculate the levodopa equivalent dose (LED), we used the formulas reported by Jost et al. [17] and the formula that a 9‐mg rotigotine patch is equivalent to a 24‐mg ropinirole patch. We calculated the LED, levodopa dose (LD), and levodopa dose divided by daily intake (LDdiv) when the patient was introduced to medical treatment and at 6, 12, 18, 24, 30, 36, 48, and 60 months after the introduction of medical treatment, and at the onset of ON/OFF fluctuations or dyskinesias. For patients without MCs, LD, LDdiv, or LED at the endpoint was defined as the dose at 24, 30, 36, 48, and 60 months after the introduction of medical treatment according to the disease course. In particular, for patients who had ON/OFF fluctuations only without dyskinesias, we calculated the medication dose at the last clinical visit.

### 2.4. Statistical Analysis

We performed statistical analyses using IBM SPSS Statistics Version 29.0.2.0. Descriptive statistics of categorical variables are reported as medians and interquartile ranges (25th–75th percentiles) and counts. We analyzed differences in sex and Hoehn and Yahr stage among groups with the chi‐square test. We estimated the incidence and median latencies of MCs, ON/OFF fluctuations, and dyskinesias by means of Kaplan–Meier survival analysis. For continuous variables, we used the Kruskal–Wallis test. For categorical variables with two groups, we used the Mann–Whitney test. For categorical variables with more than three groups, we used the Kruskal–Wallis test, followed by the Dunn–Bonferroni *post hoc* test. To compare two‐tailed categorical variables within the same patient, we used the Wilcoxon signed‐rank test. Results with two‐tailed *p* values < 0.05 were considered statistically significant.

## 3. Results

We retrospectively assessed 131 patients (63 men and 68 women) with PD. However, we excluded 11 patients who experienced falls, introducing the necessity for gait assistance, or freezing of gait, either before the first presentation or in the period in which their medications were increased in the very early stage of treatment. Thus, we analyzed 120 patients (60 men and 60 women). The characteristics of the 120 patients are shown in Table 1.

**TABLE 1:** Characteristics of the patients.

Characteristic	Value
Patients, *n* (male, female)	120 (60, 60)
Duration, months	52 (31–81)
Age at onset, years	72 (64–77)
Time from onset to treatment initiation, months	11 (3–19)
Hoehn and Yahr Stage I/II	47/73
LD at treatment initiation, mg	100 (100–150)
LDdiv at treatment initiation, mg	50 (50–50)
LED at treatment initiation, mg	142.5 (100–150)

*Note:* Data are numbers of patients, numbers, or medians (interquartile ranges).LDdiv, levodopa dose divided by daily intake.

Abbreviations: LD, levodopa dose; LED, levodopa equivalent dose.

Among the 120 patients, 76 experienced MCs, whereas 44 did not. There was no significant difference between patients with and without MCs in terms of the latency of MCs or disease duration, age at onset, time from onset to treatment initiation, Hoehn and Yahr Stage I or II at the initiation of medical treatment, and LD, LDdiv, and LED at the endpoint (Table 2).

**TABLE 2:** Comparison between patients with or without motor complications.

	Patients with MCs at onset (*n* = 76)	Patients without MCs at endpoint (*n* = 44)	*p*
Sex, male/female	35/41	25/19	ns
Duration/latency, months	28 (14–41)	32.5 (24–49)	ns
Age at onset, years	71 (63–75)	74 (66–78)	ns
Time from onset to treatment initiation, months	12 (3–24)	8 (3–17)	ns
Hoehn and Yahr Stage I/II	33/43	14/30	ns
LD, mg	300 (237.5–350)	300 (200–350)	ns
LDdiv, mg	100 (79–111)	100 (67–100)	ns
LED, mg	300 (250–450)	300 (200–399.25)	ns

*Note:* Data are numbers of patients, numbers, or medians (interquartile ranges). LDdiv, levodopa dose divided by daily intake; MCs, motor complications.

Abbreviations: LD, levodopa dose; LED, levodopa equivalent dose; ns, not significant.

**FIGURE 1:**
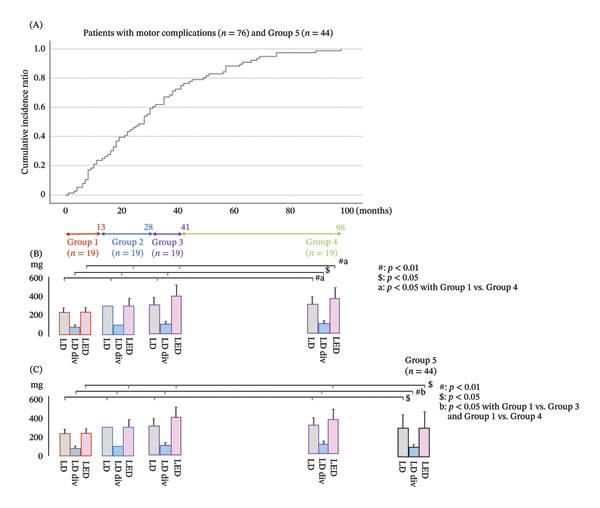
Cumulative incidence ratio curve with motor complications and medication doses. (A) Cumulative incidence curve of the onset of motor complications. (B) Medication doses in Groups 1–4. (C) Medication doses in Groups 1–5. Significant *p* values were based on the Kruskal–Wallis test and Dunn–Bonferroni *post hoc* test. Light brown boxes indicate the levodopa dose (LD), light blue boxes indicate the levodopa dose divided by daily intake (LDdiv), and light red boxes indicate the levodopa equivalent dose (LED).

A cumulative incidence ratio curve with MCs is shown in Figure 1A. The median latency was 28 (interquartile range: 13.75–41.25) months. The median LD, LDdiv, and LED were 300 (237.5–350), 100 (79–111), and 300 (250–450) mg, respectively (Table 2). Five patients experienced dyskinesias before ON/OFF fluctuations. We divided the 76 patients with MCs into four groups of 19 patients each, that is, Groups 1–4, according to latency. In Group 1, the last patient experienced MCs at 13 months. In Groups 2, 3, and 4, the last patient experienced MCs at 28, 41, and 98 months, respectively. The medication doses of each group are shown in Figure 1B and Supporting Table 1; age at onset and time from onset to treatment initiation are also shown in Supporting Table 1. Regarding age at onset and time from onset to treatment initiation, there were no significant differences among Groups 1–4. Comparisons of Groups 1‒4 showed a significant difference in the LD (*H* = 10.338, degrees of freedom [df] = 3, *p* < 0.01), LDdiv (*H* = 10.512, df = 3, *p* < 0.05), and LED (*H* = 10.954, df = 3, *p* < 0.01). A *post hoc* test showed significant differences regarding the LD (*Z* = −16.658, *p* < 0.05) and LED (*Z* = −18.895, *p* < 0.05) between Groups 1 and 4.

The patients who did not experience MCs (*n* = 44) were included in Group 5. Regarding medication dose, we calculated the LD, LDdiv, and LED at the last clinical visit and at the endpoint. The medication doses of Groups 1–5 are shown in Figure 1C and Supporting Table 2; age at onset and time from onset to treatment initiation are also shown in Supporting Table 2. Regarding age at onset and time from onset to treatment initiation, there were no significant differences among Groups 1–5. Comparisons of Groups 1‒5 showed a significant difference in the LD (*H* = 11.751, df = 4, *p* < 0.05), LDdiv (*H* = 17.259, df = 4, *p* < 0.01), and LED (*H* = 12.129, df = 4, *p* < 0.05). A *post hoc* test showed significant differences regarding the LDdiv between Groups 1 and 3 (*Z* = −32.500, *p* < 0.05) and between Groups 1 and 4 (*Z* = −31.053, *p* < 0.05).

Table 3 shows the characteristics of patients who experienced ON/OFF fluctuations only (*n* = 57) and those with dyskinesias (*n* = 19). The patients who experienced dyskinesias had a younger age at onset (*U* = 711.500, *p* < 0.05) as well as a higher LD (*U* = 217.500, *p* < 0.01), LDdiv (*U* = 271.500, *p* < 0.01), and LED (*U* = 185.500, *p* < 0.01) than those who experienced ON/OFF fluctuations only.

**TABLE 3:** Comparison between patients with ON/OFF fluctuations only and dyskinesias.

	ON/OFF fluctuations only (*n* = 57)	Dyskinesias (*n* = 19)	*p*
Sex, male/female	28/29	7/12	ns
Latency, months	28 (14–41)	50 (29–69)	< 0.01
Age at onset, years	72 (66–76)	65 (62–71)	< 0.05
Time from onset to treatment initiation, months	14 (3–21)	9 (3–24)	ns
Hoehn and Yahr Stage I/II	28/29	8/11	ns
LD at onset, mg	300 (200–300)	400 (375–500)	< 0.001
LDdiv at onset, mg	100 (67–100)	125 (105–133)	< 0.001
LED at onset, mg	300 (249–400)	600 (466–797.75)	< 0.001

*Note:* Data are numbers of patients, numbers, or medians (interquartile ranges). LDdiv, levodopa dose divided by daily intake.

Abbreviations: LD, levodopa dose; LED, levodopa equivalent dose; ns, not significant.

**FIGURE 2:**
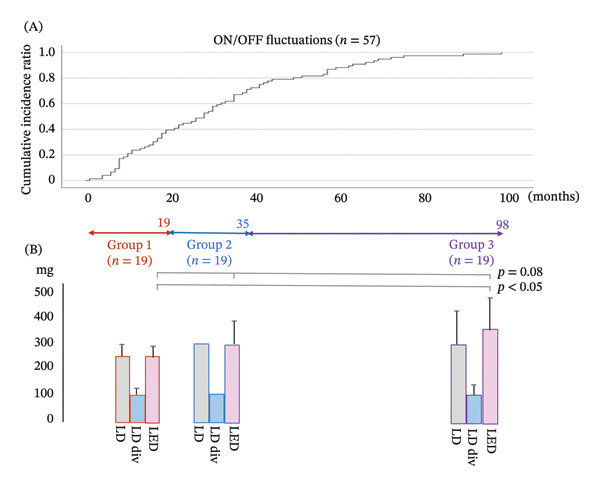
Cumulative incidence ratio curve with ON/OFF fluctuations and medication doses. (A) Cumulative incidence curve of the onset of ON/OFF fluctuations. (B) Medication doses in Groups 1–3. Significant *p* values were based on the Mann–Whitney test. Light brown boxes indicate the levodopa dose (LD), light blue boxes indicate the levodopa dose divided by daily intake (LDdiv), and light red boxes indicate the levodopa equivalent dose (LED).

A cumulative incidence ratio curve of patients who experienced ON/OFF fluctuations only is shown in Figure 2A. The median latency was 28 (13–41) months. The median LD, LDdiv, and LED were 300 (200–300), 100 (67–100), and 300 (249–400) mg, respectively (Supporting Table 3). We divided the 57 patients who experienced ON/OFF fluctuations only into three groups of 19 patients each, i.e., Groups 1–3, according to latency. In Groups 1, 2, and 3, the last patient experienced ON/OFF fluctuations at 19, 35, and 98 months, respectively. The medication doses of each group are shown in Figure 2B and Supporting Table 3; age at onset and time from onset to treatment initiation are also shown in Supporting Table 3. There was no significant difference among Groups 1–3 regarding age at onset, time from onset to treatment initiation, LD, LDdiv, and LED. However, regarding the LED, there was a significant difference between Groups 1 and 3 (*U* = 254.000, *p* < 0.05).

**FIGURE 3:**
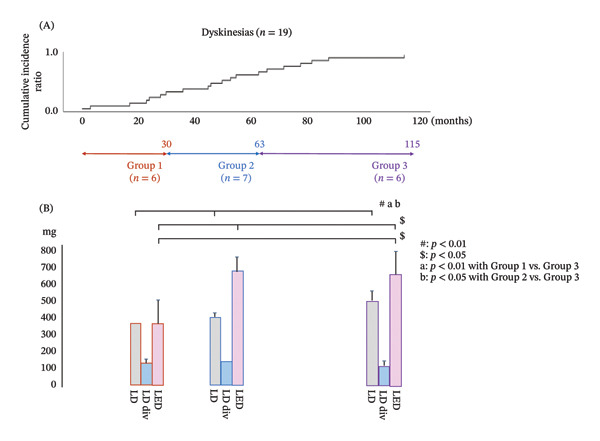
Cumulative incidence ratio curve with dyskinesias and medication doses. (A) Cumulative incidence curve of the onset of dyskinesia. (B) Medication doses in Groups 1–3. Significant *p* values were based on the Kruskal–Wallis test, Dunn–Bonferroni *post hoc* test, and Mann–Whitney test. Light brown boxes indicate the levodopa dose (LD), light blue boxes indicate the levodopa dose divided by daily intake (LDdiv), and light red boxes indicate the levodopa equivalent dose (LED).

A cumulative incidence ratio curve of 19 patients who experienced dyskinesias is shown in Figure 3A. The median latency was 50 (29–69) months. The median LD, LDdiv, and LED were 400 (375–500), 125 (105–133), and 600 (466–797.75) mg, respectively (Supporting Table 4). We divided these 19 patients into three groups according to latency: Groups 1 and 3 consisted of six patients, and Group 2 consisted of seven patients. In Groups 1, 2, and 3, the last patient experienced dyskinesias at 30, 63, and 115 months, respectively. The medication doses of each group are shown in Figure 3B and Supporting Table 4; age at onset and time from onset to treatment initiation are also shown in Supporting Table 4. There were significant differences among Groups 1–3 regarding the LD (*H* = 10.586, df = 2, *p* < 0.01) and LED (*H* = 6.223, df = 2, *p* < 0.05). A *post hoc* test showed significant differences in the LD between Groups 1 and 3 (*Z* = −9.750, *p* < 0.01) and between Groups 2 and 3 (*Z* = −7.929, *p* < 0.05). There was a significant difference in the LED between Groups 1 and 3 (*U* = 32.000, *p* < 0.05).

Among the 19 patients who showed dyskinesias, 17 showed peak‐dose dyskinesias only, while the other two patients showed both diphasic and peak‐dose dyskinesias. This result did not affect the onset of dyskinesias in this study.

Table 4 shows the characteristics and medication doses of five patients who experienced dyskinesias before ON/OFF fluctuations and 14 patients who experienced dyskinesias after ON/OFF fluctuations. There was no significant difference in medication dose at the onset of ON/OFF fluctuations between the two groups. In the five patients who experienced dyskinesias before ON/OFF fluctuations, there were no significant changes in the LD, LDdiv, and LED between the onset of ON/OFF fluctuations and the onset of dyskinesias. However, in the 14 patients who experienced dyskinesias after ON/OFF fluctuations, there were significant differences between the LD at the onset of ON/OFF fluctuations and the LD at the onset of dyskinesias (*T* = 78.000, *p* < 0.01), LDdiv at the onset of ON/OFF fluctuations and LDdiv at the onset of dyskinesias (*T* = 67.000, *p* < 0.05), and LED at the onset of ON/OFF fluctuations and LED at the onset of dyskinesias (*T* = 90.000, *p* < 0.01).

**TABLE 4:** Characteristics and medication doses of patients who experienced dyskinesias before ON/OFF fluctuations and patients who experienced dyskinesias after ON/OFF fluctuations.

	Dyskinesias before ON/OFF fluctuations (*n* = 5)	Dyskinesias after ON/OFF fluctuations (*n* = 14)	*p*
Sex, male/female	1/4	6/8	ns
Age at onset, years	62 (56–71)	66 (62–69)	ns
Time from onset to treatment initiation, months	10 (7–24)	9 (2–23)	ns
Hoehn and Yahr Stage I/II	1/4	6/8	ns
Onset of dyskinesias, months	28 (24–45)	59 (38.5–76.5)	< 0.001
LD at onset of dyskinesias, mg	300 (300–400)	450 (400–500)	< 0.05
LDdiv at onset of dyskinesias, mg	100 (100–133)	125 (113–133)	ns
LED at onset of dyskinesias, mg	400 (300–400)	687.5 (600–962.625)	< 0.01
Onset of ON/OFF fluctuations, months	33 (26–59)	26.5 (814.5–38)	ns
LD at onset of ON/OFF fluctuations, mg	300 (300–350)	300 (300–350)	ns
LDdiv at onset of ON/OFF fluctuations, mg	100 (100–117)	100 (100–117)	ns
LED at the onset of ON/OFF fluctuations, mg	350 (300–478)	399 (300–546.9)	ns

*Note:* Data are numbers of patients, numbers, or medians (interquartile ranges). LDdiv, levodopa dose divided by daily intake.

Abbreviations: LD, levodopa dose; LED, levodopa equivalent dose; ns, not significant.

Finally, in Table 5, we compared sex, age at onset, disease duration, latency, LD, LDdiv, and LED in 57 patients who experienced ON/OFF fluctuations only at the last visit and in 19 patients who experienced dyskinesias. Although the patients who experienced ON/OFF fluctuations only at the last visit had a higher age at onset than those who experienced dyskinesias (*H* = 5.344, df = 1, *p* < 0.05), there was no significant difference in sex, disease duration, latency for dyskinesias, and medication dose.

**TABLE 5:** Comparison between patients with ON/OFF fluctuations only at the last visit and patients with dyskinesias at onset.

	ON/OFF fluctuations only at last visit (*n* = 57)	Dyskinesias at onset (*n* = 19)	*p*
Sex, male/female	28/29	7/12	ns
Age at onset, years	72 (66–76)	65 (62–71)	< 0.05
Duration/latency, months	65 (48–82)	50 (29–69)	ns
LD, mg	400 (300–450)	400 (375–500)	ns
LDdiv, mg	117 (100–138)	125 (111.25–133)	ns
LED, mg	550 (400–702.5)	600 (466–822.75)	ns

*Note:* Data are numbers of patients, numbers, or medians (interquartile ranges); LDdiv, levodopa dose divided by daily intake.

Abbreviations: LD, levodopa dose; LED, levodopa equivalent dose; ns, not significant.

## 4. Discussion

Among 120 patients with PD (Hoehn and Yahr Stage I or II; median age at onset, 72 years), 76 experienced MCs with a median latency of 28 months, whereas 44 did not experience MCs by the end of the study period. The latency and medication dose at onset with MCs varied (Figure 1 and Supporting Tables 1 and 2). There were no significant differences in the LD, LDdiv, and LED between patients with and without MCs (Table 2). As patients with more severe motor disabilities experience motor fluctuations or wearing‐off earlier [9, 12], we excluded patients who experienced falls requiring the introduction of gait assistance or freezing of gait before the first presentation or in the very early stage of treatment.

In the analysis of Groups 1–5, Group 1 had a lower medication dose than Group 5, which did not experience MCs. In addition, Group 5 had a similar latency and medication dose to those of Groups 2 and 3. These results suggest that the medication dose in each patient is not the sole cause of MCs, and there must be other factors that lead to their development.

We also analyzed patients who developed ON/OFF fluctuations and those who experienced dyskinesias. The patients who experienced dyskinesias had a significantly younger age at disease onset and higher medication dose than those who developed ON/OFF fluctuations (Table 3). Regarding ON/OFF fluctuations, a significant difference was only observed in the LED between the shortest and longest latency groups (Figure 2 and Supporting Table 3). Regarding dyskinesias, the longest latency group had a significantly higher LD than the other two groups, and there was a significant difference in the LED between the shortest and longest latency groups (Figure 3 and Supporting Table 4).

In terms of the analysis of medication doses throughout the study, namely any of the MCs, ON/OFF fluctuations, or dyskinesias, we consistently found that the group with the shortest latency received the lowest medication dose, whereas the group with the longest latency had the highest medication dose.

Regarding the occurrence of motor fluctuations and dyskinesias in PD, several observations have been reported. The Parkinson Study Group found that the cumulative LD and LED were associated with an earlier occurrence of motor fluctuations and dyskinesias [11]. Olanow et al. for the STRIDE‐PD investigators [12] reported that a lower age at onset, higher Movement Disorder Society‐sponsored revision of the Unified PD Rating Scale Part III score, living in North America, higher LD, and being female were predictive factors of the time to wearing‐off. Conversely, lower age at onset, higher LD, living in North America, lower body weight, L‐dopa/carbidopa/entacapone administration, being female, and higher Movement Disorder Society‐sponsored Revision of the Unified PD Rating Scale Part III score were predictive factors of the time to dyskinesias. Wearing‐off occurred first in 33% of patients, whereas dyskinesias occurred first in 19%. Therefore, the higher the LD, the higher the frequency of patients developing both wearing‐off and dyskinesias. These studies reported the cumulative incidence ratio regarding any motor fluctuation, ON/OFF fluctuations, or dyskinesias.

Regarding the medication dose, in the study conducted by the Parkinson Study Group [11], the subjects were randomized in a blinded manner, and there was a dose escalation phase. In the STRIDE‐PD study [12], the subjects were followed in a blinded manner and were required to take medications four times daily at 3.5‐h intervals. The medication dose could be decreased or increased. Different from these studies, our study was neither randomized nor blinded, and there was no restriction on medication frequency per day [12].

In the Ghana study [13], at a median of 1 year after the introduction of levodopa treatment, the prevalence of motor fluctuations and dyskinesias was 56% (90.0% were wearing off) and 14%, respectively. Similar to our study, levodopa was slowly titrated up and kept at a relatively low dose. Median disease duration at the first appearance of motor fluctuations and dyskinesias in Ghanaian patients was 6.0 years. The median duration of levodopa therapy at the onset of motor fluctuations and dyskinesias was 0.5 and 1.0 years, respectively. Similar to our study, the Ghana study was not randomized or blinded, and there was no restriction on medication frequency per day. The authors did not report the mean LD at the onset of motor fluctuations, but did state that the mean LD at the onset of dyskinesias was 365 mg. None of their patients were receiving concomitant dopamine agonist therapy.

Conversely, we reported the cumulative incidence of ON/OFF fluctuations, showing a wide variation in each patient, with a median latency of 28 months. Regarding the LED, although there was a wide variation, the group with the shortest latency was under the lowest LED, whereas the group with the longest latency was under the highest LED. Furthermore, the authors of the Ghana study suggested that the primary cause of the early appearance of MC was disease progression rather than a high medication dose [13]. As the median LED in patients in the shortest latency group who experienced ON/OFF fluctuations was 250 mg in our study, this is in line with the results from the Ghana study [13].

Although neuronal loss in the substantia nigra pars compacta and the subsequent depletion of synaptic dopamine are important pathophysiological factors in levodopa‐induced MCs, the pathophysiology of ON/OFF fluctuations in the striatum has not been well documented. Presynaptic impaired dopamine storage due to dopamine transporter function failure is the most accepted theory [18]. Furukawa et al. [19] used ^123^I‐ioflupane single photon emission computed tomography and neuromelanin‐sensitive magnetic resonance imaging and reported that in patients with early PD (motor symptoms for ≤ 8 or 10 years), motor dysfunction during the drug‐off state was paralleled by a decline in ^123^I‐ioflupane uptake in the striatum, while in patients with advanced PD (motor dysfunction for > 8 or 10 years and with fluctuations), the motor deficit during the drug‐off state negatively correlated with neuromelanin‐sensitive magnetic resonance imaging signals in the substantia nigra. Thus, disease progression differentially affects the nigrostriatal pathway, that is, the pathological changes result in the occurrence of ON/OFF fluctuations.

Finally, there were two more important observations in our study. First, we found that five patients experienced dyskinesias before ON/OFF fluctuations. These five patients and 14 other patients who experienced dyskinesias after ON/OFF fluctuations were under similar medication doses at the onset of the ON/OFF fluctuations. At the onset of dyskinesias, the five patients were under a significantly lower LD and LED than the other 14 patients. In addition, the LD, LDdiv, and LED of the five patients at the onset of ON/OFF fluctuations and dyskinesias were similar. Therefore, the pathophysiological background for ON/OFF fluctuations in these five patients and the 57 patients who experienced ON/OFF fluctuations only (Table 3) may be different. Furthermore, the pathophysiological background for dyskinesias in the five patients who experienced dyskinesia before ON/OFF fluctuations and the 14 patients who experienced dyskinesia after ON/OFF fluctuations may be different, as the five patients did not need a higher medication dose for dyskinesias to develop.

Second, regarding the patients who experienced ON/OFF fluctuations, but have not experienced dyskinesias yet, even after the medication dose was increased to a comparable level as administered to patients who experienced dyskinesias (Table 5). The patients who experienced dyskinesias had a significantly younger age at onset than those who experienced ON/OFF fluctuations but had not experienced dyskinesias yet. However, both groups had comparable disease duration or the latency to the onset of dyskinesias and medication doses. It has been generally accepted that when patients experience dyskinesias after ON/OFF fluctuations, the pathophysiological changes and higher medication dose on top of the background of ON/OFF fluctuations lead to dyskinesias [20, 21]. However, our results show that a higher medication dose does not always lead to dyskinesias.

Pellecchia et al. reported that being female is the main predictor of wearing‐off and dyskinesias in levodopa‐naïve patients with PD. In their study, age at onset was 63.81 years in men and 65.34 years in women. They suggested that older age was a significant protective factor [22]. In contrast, in our study, age at onset was 72 years. In addition, there was no significant sex difference between patients with or without MCs (Table 2), as well as between patients with ON/OFF only or dyskinesias (Table 3). Therefore, older age at onset may be able to explain why sex was not a predictor of dyskinesia in this study.

Regarding the difference in the pathophysiological background of dyskinesias, Radojević et al. [23] reported that genetic variants, particularly in the *COMT* and *ANKK1*/*DRD2* loci, may contribute to the development of levodopa‐induced MCs in PD. Patients with *PINK1*‐related young‐onset PD and *PRKN*‐related early‐onset PD tend to have severe dyskinesias. The median age at onset for *PINK1*‐related young‐onset PD is 32 years old, and the prevalence of motor fluctuations or dyskinesias is 79% or 67%, respectively [24]. The median age at onset for *PRKN*‐related early‐onset PD is 31 years old, and the prevalence of motor fluctuations or dyskinesias is 21% or 24%, respectively. A marked and sustained response to levodopa, which is frequently associated with levodopa‐induced motor fluctuations and dyskinesias, has been reported [25]. However, in the present study, the median age at onset in patients who experienced dyskinesias was 65 years (interquartile range, 62–71 years). Although we did not perform genetic analysis, only a small portion of the patients who showed dyskinesias could have either a *PINK1* or a *PRKN* variant.

This study may have limitations in terms of generalization. Furthermore, the results could be biased because of the inclusion of relatively old patients and the study’s retrospective and single‐center design. In addition, we did not use the Movement Disorders Society Unified Dyskinesia Rating Scale, and only some of the included patients underwent ^123^I‐ioflupane single photon emission computed tomography.

## 5. Conclusions

In conclusion, in patients with PD, there was a wide variation in the latencies of MCs, ON/OFF fluctuations, and dyskinesias in each patient. In addition, there was a wide variation in medication dose at the onset of ON/OFF fluctuations and dyskinesias. At the onset of ON/OFF fluctuations and dyskinesias, the patients in the shortest latency group were under the lowest medication dose, whereas those in the longest latency group were under the highest medication dose. There were patients who experienced dyskinesias before ON/OFF fluctuations. Furthermore, there were patients who did not experience dyskinesias even with higher medication doses that were higher than those of patients who experienced dyskinesias after they experienced ON/OFF fluctuations, and a younger age at onset may be associated with the occurrence of dyskinesias in patients who have already experienced ON/OFF fluctuations. Thus, the pathophysiological backgrounds and changes causing ON/OFF fluctuations and dyskinesias are not the same for all patients with PD.

## Author Contributions

Yasushi Osaki organized and conceptualized the design, executed the study, analyzed the data, and wrote and edited the final version of the manuscript. Yukari Morita organized and executed the design, executed the study, and edited the final version of the manuscript. Sho Ohtsuru, Tomohiro Shogase, Daiji Yoshimoto, Tatsuya Ikeda, Sayomi Kabeya, and Yu Hashimoto executed the study and edited the final version of the manuscript. Takuya Matsushita conceptualized the study and edited the final version of the manuscript.

## Funding

No funding was received for this work.

## Conflicts of Interest

Yasushi Osaki received honoraria from Chugai Pharmaceutical, Biogen Japan, and Fujimoto Pharmaceutical Corporation, unrelated to this research. Yukari Morita received honoraria from Biogen Japan, Ono Pharmaceuticals, and Argenx Japan K.K., unrelated to this research. The other authors declare no conflicts of interest.

## Supporting Information

Additional supporting information can be found online in the Supporting Information section.

## Supporting information


**Supporting Information** Supporting materials include: Supporting Figure 1: Flow diagram of the study; Supporting Table 1: Age at onset, time from onset to treatment initiation, and medication doses in Groups 1–4; Supporting Table 2: Age at onset, time from onset to treatment initiation, and medication doses in Groups 1–5; Supporting Table 3: Age at onset, time from onset to treatment initiation, and medication doses in patients who experienced ON/OFF fluctuations; Supporting Table 4: Medication doses in patients who experienced dyskinesias; and STROBE checklist.

## Data Availability

The data that support the findings of this study are available from the corresponding author upon reasonable request.
