# Supplementary material for: Onset of Motor Complications and Medication Dose in Newly Diagnosed and Treated Parkinson’s Disease
Source: Parkinsons Dis. 2026 Jul 31;2026:8827381. doi: 10.1155/padi/8827381 (PMC13424818; doi:10.1155/padi/8827381)
Supplement: Supplementary file 1 — Supporting Information Supporting materials include: Supporting Figure 1: Flow diagram of the study; Supporting Table 1: Age at onset, time from onset to treatment initiation, and medication doses in Groups 1–4; Supporting Table 2: Age at onset, time from onset to treatment initiation, and medication doses in Groups 1–5; Supporting Table 3: Age at onset, time from onset to treatment initiation, and medication doses in patients who experienced ON/OFF fluctuations; Supporting Table 4: Medication doses in patients who experienced dyskinesias; and STROBE checklist. [file PADI-2026-8827381-s001.zip › Supplementary Fig & Tables Padi Osaki rev black & white.docx]

**Suppl. Figure 1. Flow diagram of the study**

**Suppl. Table 1. Age at onset, time from onset to treatment initiation, and medication doses in Groups 1‒4**

|  | **Group 1 (*n* = 19)** | **Group 2 (*n* = 19)** | **Group 3 (*n* = 19)** | **Group 4 (*n* = 19)** | ***P*** |
| --- | --- | --- | --- | --- | --- |
| Age at onset, years | 73 (68‒80) | 71 (64‒74) | 70 (63‒85) | 66 (62‒74) | ns |
| Time from onset to treatment initiation, months | 10 (2‒28) | 14 (8‒22) | 8 (1‒17) | 15 (9‒23) | ns |
| LD, mg | 250 (150‒300) | 300 (200‒300) | 300 (300‒375) | 300 (300‒375) | <0.01* |
| LDdiv, mg | 83 (50‒100) | 100 (67‒100) | 100 (100‒121) | 100 (100‒125) | <0.05 |
| LED, mg | 250 (175‒300) | 300 (225‒374.5) | 399 (300‒531) | 360 (300‒527) | <0.05* |

Data are numbers of patients, numbers, or median (interquartile range). Kruskal-Wallis test followed by the Dunn-Bonferroni *post* *hoc* test for multiple sample testing. LD, levodopa dose; LDdiv, levodopa dose divided by daily intake; LED, levodopa equivalent dose; ns, not significant. **P* < 0.05 between Groups 1 and 4.

**Suppl. Table 2. Age at onset, time from onset to treatment initiation, and medication doses in Groups 1‒5**

|  | **Group 1**  **(*n* = 19)** | **Group 2**  **(*n* = 19)** | **Group 3**  **(*n* = 19)** | **Group 4**  **(*n* = 19)** | **Group 5**  **(*n* = 44)** | ***P*** |
| --- | --- | --- | --- | --- | --- | --- |
| Age at onset, years | 73 (68‒80) | 71 (64‒74) | 70 (63‒85) | 66 (62‒74) | 74 (66‒78) | ns |
| Time from onset to treatment initiation, months | 10 (2‒28) | 14 (8‒22) | 8 (1‒17) | 15 (9‒23) | 8 (3‒17) | ns |
| LD, mg | 250 (150‒300) | 300 (200‒300) | 300 (300‒375) | 300 (300‒375) | 300 (200‒350) | <0.05 |
| LDdiv, mg | 83 (50‒100) | 100 (67‒100) | 100 (100‒121) | 100 (100‒125) | 95 (67‒100) | <0.01* |
| LED, mg | 250 (175‒300) | 300 (225‒374.5) | 399 (300‒531) | 360 (300‒527) | 300 (200‒399.25) | <0.05 |

Data are numbers of patients, numbers, or median (interquartile range). Kruskal-Wallis test followed by the Dunn-Bonferroni *post* *hoc* test for multiple sample testing. LD, levodopa dose; LDdiv, levodopa dose divided by daily intake; LED, levodopa equivalent dose. **P* < 0.05 between Groups 1 and 3, and between Groups 1 and 4.

**Suppl. Table 3. Age at onset, time from onset to treatment initiation, and medication doses in patients who experienced ON/OFF fluctuations**

|  | **Total (*n* = 57)** | **Groups** | | | **Comparison** |
| --- | --- | --- | --- | --- | --- |
|  |  | **Group 1 (*n* = 19)** | **Group 2 (*n* = 19)** | **Group 3 (*n* = 19)** | ***P*** |
| Age at onset, years | 72 (65‒76) | 74 (68‒80) | 72 (61‒75) | 73 (63‒75) | ns |
| Time from onset to treatment initiation, months | 14 (3‒21) | 12 (3‒25) | 6 (2‒16) | 15 (12‒24) | ns |
| LD, mg | 300 (200‒300) | 250 (175‒300) | 300 (225‒300) | 300 (300‒425) | ns |
| LDdiv, mg | 100 (67‒100) | 83 (58‒100) | 100 (75‒100) | 100 (100‒142) | ns |
| LED, mg | 300 (249‒400) | 250 (200‒300)* | 300 (250‒399.5) | 350 (300‒482.5)* | 0.08 |

Data are numbers of patients, numbers, or median (interquartile range). Kruskal-Wallis test. LED, levodopa equivalent dose; LDdiv, levodopa dose divided by daily intake; LD, levodopa dose. **P* < 0.05 between Groups 1 and 3 by Mann-Whitney test.

**Suppl. Table 4. Medication doses in patients who experienced dyskinesias**

|  | **Total (*n* = 19)** | **Groups** | | | **Comparison** |
| --- | --- | --- | --- | --- | --- |
|  |  | **Group 1 (*n* = 6)** | **Group 2 (*n* = 7)** | **Group 3 (*n* = 6)** | ***P*** |
| Age at onset, years | 65 (62‒71) | 73 (69‒76) | 62 (59‒67) | 63 (62‒66) | ns |
| Time from onset to treatment initiation, months | 10 (4‒24) | 9 (4‒11) | 24 (4‒34) | 9 (3‒16) | ns |
| LD, mg | 400 (375‒500) | 350 (225‒400) | 400 (375‒425) | 525 (500‒550) | <0.01* § |
| LDdiv, mg | 125 (105‒133) | 113 (75‒131) | 133 (115‒133) | 119 (111‒134) | ns |
| LED, mg | 600 (466‒797.75) | 350 (225‒499) ^¶^ | 670 (569.5‒710.25) | 685 (617.5‒876.25) ^¶^ | <0.05 |

Data are numbers of patients, numbers, or median (interquartile range). Kruskal-Wallis test followed by the Dunn-Bonferroni *post* *hoc* test for multiple sample testing LD, levodopa dose; LDdiv, levodopa dose divided by daily intake; LED, levodopa equivalent dose. **P* < 0.01 between Groups 1 and 3. ^§^*P* < 0.05 between Groups 2 and 3. ^¶^*P* < 0.05 between Groups 1 and 3 by Mann-Whitney test.
